# Detection of visible-wavelength aurora on Mars

**DOI:** 10.1126/sciadv.ads1563

**Published:** 2025-05-14

**Authors:** Elise W. Knutsen, Timothy H. McConnochie, Mark Lemmon, Chris Donaldson, Raymond Francis, Carey Legett, Shayla B. Viet, Lauriane Soret, Daniel Toledo, Victor Apéstigue, Olivier Witasse, Franck Montmessin, Rebecca Jolitz, Nicolas M. Schneider, Leslie Tamppari, Agnès Cousin, Roger C. Wiens, Sylvestre Maurice, James F. Bell, Olivier Forni, Jeremie Lasue, Paolo Pilleri, Tanguy Bertrand, Priya Patel, Susanne Schröder, Shannon Curry, Christina O. Lee, Ali Rahmati

**Affiliations:** ^1^ University of Oslo, CENSSS, Oslo, Norway.; ^2^ Space Science Institute, Boulder, CO, USA.; ^3^ Malin Space Science Systems, San Diego, CA, USA^.^; ^4^ Jet Propulsion Laboratory, California Institute of Technology, Pasadena, CA, USA.; ^5^ Los Alamos National Laboratory, Los Alamos, NM, USA.; ^6^ Norwegian University of Science and Technology, Trondheim, Norway.; ^7^ Laboratoire de Physique Atmosphérique et Planétaire, STAR Institute, Université de Liège, Liege, Belgium.; ^8^ Instituto Nacional De Técnica Aerospacial (INTA), Madrid, Spain.; ^9^ ESA/ESTEC, Noordwijk, Netherlands.; ^10^ LATMOS/IPSL, UVSQ Université Paris-Saclay, Sorbonne Université, CNRS, Paris, France.; ^11^ Laboratory for Atmospheric and Space Physics, University of Colorado, Boulder, CO, USA.; ^12^ IRAP–Institut de recherche en astrophysique et planétologie, Toulouse, France.; ^13^ Purdue University, West Lafayette, IN, USA.; ^14^ School of Earth and Space Exploration, Arizona State University, Tempe, AZ, USA.; ^15^ LESIA, Paris Observatory, Paris, France.; ^16^ Mullard Space Science Laboratory, University College London, London, England.; ^17^ Deutsches Zentrum für Luft- und Raumfahrt (DLR), Institute of Optical Sensor Systems, Berlin, Germany.; ^18^ Space Sciences Laboratory, University of California, Berkeley, CA, USA.

## Abstract

Mars hosts various auroral processes despite the planet’s tenuous atmosphere and lack of a global magnetic field. To date, all aurora observations have been at ultraviolet wavelengths from orbit. We describe the discovery of green visible-wavelength aurora, originating from the atomic oxygen line at 557.7 nanometers, detected with the SuperCam and Mastcam-Z instruments on the Mars 2020 Perseverance rover. Near–real-time simulations of a Mars-directed coronal mass ejection (CME) provided sufficient lead-time to schedule an observation with the rover. The emission was observed 3 days after the CME eruption, suggesting that the aurora was induced by particles accelerated by the moving shock front. To our knowledge, detection of aurora from a planetary surface other than Earth has never been reported, nor has visible aurora been observed at Mars. This detection demonstrates that auroral forecasting at Mars is possible, and that during events with higher particle precipitation, or under less dusty atmospheric conditions, aurorae will be visible to future astronauts.

## Teaser

Green aurora lit up the martian sky following a solar storm eruption, captured by instruments on the Mars 2020 Perseverance rover.

## INTRODUCTION

Planetary aurorae have been observed on every planet with an atmosphere in our Solar System. In response to the precipitation of energetic particles, emissions span x-ray to radio wavelengths and can be used to constrain the incoming particle energy distribution (*1*–*3*) and as a means of remotely sensing the conditions of the emission source region (*4*, *5*).

Mars is, as far as we know, the only planet in our Solar System with a hybrid magnetosphere, with elements of both the induced magnetosphere originating from the interplanetary magnetic field (IMF) lines being draped around Mars’ conductive ionosphere and regions of crustal magnetization (*6*, *7*) mostly confined to the ancient southern highlands (*8*). This composite field structure creates a complex magnetic morphology leading to diverse auroral phenomena, where questions of solar particle injection, precipitation, and transport remain open, particularly with respect to magnetotail instabilities and acceleration mechanisms and the role of magnetic topology and magnetic reconnection (*9*–*11*).

Several morphologies of aurorae have so far been observed on Mars: localized discrete and patchy aurora (*12*, *13*), global diffuse aurora (*14*), dayside proton aurora (*15*), and large-scale sinuous aurora (*10*, *16*). These can be categorized by the precipitating particle energies, namely, solar wind aurora (proton aurora), suprathermal aurora (discrete, sinuous aurora and patchy aurora, with electron energies of tens to hundreds of electron volts (*10*, *17*, *18*), and charged solar energetic particle (SEP) aurora (diffuse aurora). SEP and suprathermal aurora are observed intermittently on the martian nightside. Confined discrete aurorae appear frequently but are short-lived [tens of minutes to hours (*16*, *19*) and are mostly observed near the crustal fields (*18*, *19*)], while the patchy aurorae appear in nonmagnetized regions as well and often in the north where there are no crustal fields. The recently discovered sinuous aurora appears in 3% of observations from the Emirates Mars Mission Emirates Mars Ultraviolet Spectrograph and is believed to indicate a highly dynamic magnetotail (*10*, *16*). Diffuse aurorae are strongly correlated with SEP events (*20*, *21*) and can be forecasted whether the solar event is identified shortly after eruption.

SEPs are accelerated by two main processes: magnetic reconnection processes during solar flare activity (impulsive SEP events) and wave acceleration by coronal mass ejection (CME)–driven shocks (gradual SEP events) (*22*, *23*). Impulsive events accelerate the particles near the Sun, while gradual events accelerate particles as the CME-driven shock front propagates radially through the Solar System (*24*). SEPs travel along the Parker spiral (*25*) and can thus be detected at Mars if the planet is magnetically connected to a local acceleration site (at the Sun for impulsive events or to the moving shock source for gradual events). The travel time of impulsive SEPs to Mars is on the order of a few tens of minutes (for relativistic electrons) to hours (e.g., for ∼1-MeV protons) (*26*, *27*). Meanwhile, the travel time of gradual SEPs depends on when Mars is magnetically connected to the shock source as the interplanetary CME (ICME) propagates outward. If the ICME is Mars directed, then an impact can occur within 3 days after the initial eruption for a fast ICME (e.g., >1000 km/s) (*28*). The durations of impulsive and gradual SEP events vary; impulsive events can last for a few hours, while gradual events can disturb the planetary environment for several days (*22*, *29*).

Mars’ magnetosphere largely deflects and decelerates solar wind particles, but SEPs (and particularly higher-energy (a few mega–electron volts to ~100 MeV) protons (*30*) penetrate deep into the martian atmosphere [e.g., (*29*–*31*)]. The material pile-up in front of the leading shock of ICMEs can disturb the fields around Mars sufficiently to increase coupling between Mars’ magnetosphere and atmosphere, allowing for the injection of a large flux of particles, subsequently leading to auroral emissions (*32*). Using a Monte Carlo Particle TRansport In Planetary atmospheres model, Nakamura *et al.* (*33*) demonstrated that the auroral peak emission altitude varies depending on the precipitating particle species; proton-induced diffuse aurorae being both lower and brighter than electron-induced aurorae. Theoretical work has shown that 100-keV electrons are capable of inducing auroral emission at 60-km altitude (*21*, *34*), yet models predict that only a small fraction of the SEP electrons (energies in the 10- to 200-keV range) penetrates into the atmosphere (*30*). It has been hypothesized that an interplanetary disturbance, caused, for example, by ICMEs, might be required to increase the access of external particles to the atmosphere (*32*).

SEP-driven aurorae have so far been observed to be long-lasting (timescale of days) and cover the entire nightside disk of Mars (*14*, *21*). The correlation between the impact of solar events at Mars and the appearance of SEP aurora is well established (*14*, *21*), but individual events have not previously been targeted in advance for the purpose of auroral detection. Gradual, proton-dominated ICME SEP events are ideal for this purpose; we therefore designed our observation strategy around targeting diffuse SEP aurora driven by ICME-related events.

All previous aurora observations have been made from orbit and have led to the identification of several emission lines in the ultraviolet (UV) domain. The dominant lines are the CO Cameron, CO_2_^+^ UV doublet, the Fox-Duffendack-Barker (FDB) bands, and the far-UV oxygen emissions at 130 and 135 nm. The less intense atomic oxygen O(^1^S-^3^P) line at 297.2 nm is also often detected (*12*, *14*, *16*, *18*). It shares the same upper state as the 557.7-nm green line [O(^1^S → ^1^D)], which has been observed in photodissociation-induced martian dayglow (*35*, *36*). The 557.7-nm line is predicted to be present in aurorae (*18*, *37*) but has so far never been observed on Mars’ nightside and has never been observed in response to space weather events at Mars. On Venus, it has been found in nightside emissions (*38*), and the appearance correlated well with the impact of solar wind disturbances (*39*). Fainter blue and red aurora on Mars produced from the visible end of the FDB (around 400 nm) bands and the O(^1^D → ^3^P) atomic oxygen doublet (630 to 636.4 nm), respectively, were first predicted by Lilensten *et al.* (*37*). At that time, only discrete suprathermal aurora had been detected, so they (*37*) estimated an emission altitude of around 140 km, coinciding with the observed UV suprathermal aurora. The visible diffuse SEP aurora targeted here is expected to exhibit an altitude distribution similar to that of the UV SEP aurora reported in (*14*, *21*).

Despite the large number of visible-wavelength instruments at Mars, few have the necessary combination of high spectral resolution, low-light sensitivity, and scheduling flexibility to reliably detect transient auroral events. Surface assets are more limited in terms of power, data transfer, and geographic coverage compared to orbiters, but the dynamic nature of rover planning and operations allows for the implementation of a more reactive observation strategy that can take advantage of near–real-time propagation analysis of ICME events. Starting in May 2023, we made several attempts to react to such simulated SEP events and to observe with the Mars 2020 Perseverance rover instruments at times when the likelihood of auroral emission was highest. The fourth such attempt, in March 2024, yielded the first detection of visible-wavelength aurorae. We report on that detection here.

## INSTRUMENTS AND METHODS

### Solar storm selection

Several solar wind parameters can be used to gauge the local space weather conditions at Mars. Sudden increases in ion density and temperature can indicate the presence of ICME-driven SEPs (*40*), and IMF strength and the solar wind dynamic pressure are considered to be important factors and possible indicators of imminent auroral activity (*10*, *32*, *41*, *42*). To trigger diffuse aurora, kilo–electron volt–mega–electron volt SEPs are required (*14*, *33*, *34*). We focus here on gradual SEP events, as impulsive SEPs arrive at Mars too fast to plan responsive observations and since CMEs are routinely analyzed and simulated by the Moon to Mars (M2M) Space Weather Analysis Office (https://science.gsfc.nasa.gov/674/m2m/) at the NASA Goddard Space Flight Center, supported by the heliospheric modeling carried out by the NASA Community Coordinated Modeling Center (CCMC). This publicly available information is crucial in the planning of these targeted observations.

We use the NASA CCMC Space Weather Database Of Notifications, Knowledge, Information (DONKI) (https://kauai.ccmc.gsfc.nasa.gov/DONKI) to evaluate ongoing Mars-directed ICME events. DONKI provides near–real-time simulation results generated from the solar corona-solar wind model, Wang-Sheeley-Arge (WSA)–ENLIL (*43*–*45*), together with the geometric “cone” CME tool (*46*). The WSA-ENLIL model provides a time-dependent description of the background solar wind and interplanetary magnetic field from the Sun out to 2 astronomical units (AU), while the cone tool provides estimates for the initial CME parameters (angular width, initial speed at 21.5 solar radii, and solar source latitude and longitude). To plan ahead for SEP aurora observation campaigns, we monitor Mars-facing solar active regions and routinely review DONKI for new Mars-directed ICME event simulations. For the particular event reported here, we were notified of the ICME activity through the Mars Space Weather Alert Notification email distributed to several operational Mars mission projects, courtesy of the Mars Atmosphere and Volatile Evolution (MAVEN) mission.

**Fig. 1.**
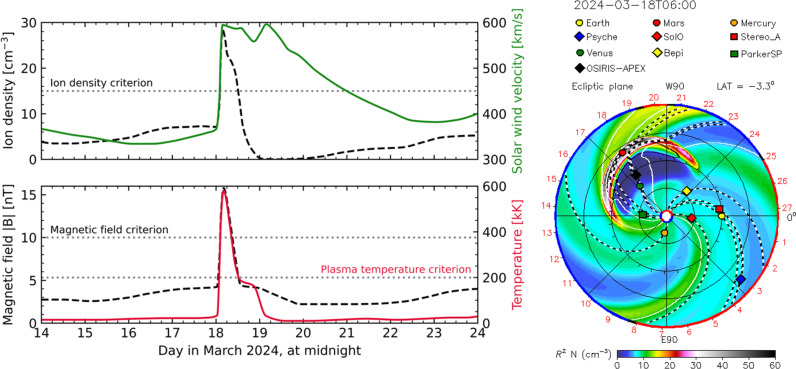
Simulation of the solar wind plasma and IMF enhancement at Mars during the ICME event. The figure is adapted from simulation results generated by the NASA CCMC using the WSA-ENLIL + cone model. The left figure shows the solar wind ion density and velocity in the top and the IMF magnitude and plasma temperature in the bottom, with horizontal dotted lines marking the corresponding solar event selection criteria as described in the “Solar storm selection” section. The right figure shows a time snapshot of the modeled solar wind ion density (scaled by solar distance squared) in the ecliptic plane from 0.1 to 2 AU at the time of peak ion density at Mars, coinciding with the maximum compression of the solar wind at 1.5 AU by the ICME structure. The full simulation and solar activity details are available at https://kauai.ccmc.gsfc.nasa.gov/DONKI/view/WSA-ENLIL/29602/1.

To initiate an aurora rover observation, we evaluate the simulated ICME properties from the CCMC simulation and target ICME events that fulfill certain requirements. These requirements were initially based on statistical solar wind properties at Mars (*47*) and by studying previous Mars-impacting ICMEs. After two unsuccessful attempts, the criteria were refined to increase the chances of auroral detection. The criteria used for this detection (visualized in Fig. 1) were as follows: an initial CME ejection velocity > 800 km/s estimated from white light solar coronagraph imaging, modeled values of ion density > 15 ions/cm^3^, IMF strength > 10 nT, and plasma temperature > 2 × 10^5^ K at Mars.

When an ICME fulfilling the criteria listed above is identified, we attempt to schedule an observation with the Perseverance rover’s SuperCam and Mastcam-Z instruments as close in time as possible after the predicted peak time of the solar wind ion density. Note that it is not always possible to schedule an observation for a selected ICME due to a combination of factors: limited rover resources, timing of the event simulations posted to DONKI, timing of when or whether the Mars space weather alert notifications are issued, or the estimated ICME arrival time at Mars might align poorly with the martian day/night and rover planning cycles.

### Observation strategy

The Mars 2020 Perseverance rover (*48*) landed in Jezero crater on 18 February 2021, at 18.5° north and 77° east longitude. For timekeeping purposes, each martian day is numbered, with mission sol 0 being the day of landing. Of its seven instruments, SuperCam and Mastcam-Z have the best combination of sensitivity and operational flexibility to detect diffuse aurora. The Radiation and Dust Sensor (RDS) (*49*), which is part of the Mars Environmental Dynamics Analyzer (MEDA) instrument suite (*50*), covers the wavelengths of UV auroral lines, but since RDS sensitivity was optimized for solar flux, its chances of detecting the weaker UV emission are low. The rover observation strategy includes acquisition of visible spectra with SuperCam and color images with Mastcam-Z, with instrument settings tailored to our assumptions on the appearance of diffuse aurora from the surface.

#### Perseverance SuperCam

SuperCam is a multitechnique spectrometer designed for laser-induced breakdown (LIBS) and Raman spectroscopy and visible-infrared reflectance spectroscopy (*51*, *52*). Only the passive mode at wavelengths below 1 μm is relevant to aurora observations. SuperCam’s visible-range spectrometer covers the 535- to 853-nm range, with a spectral resolution of 0.35 nm full width at half maximum (FWHM) in the green spectral range and 0.6 nm FWHM in the “orange” channel (which includes the 630-nm red emission line). SuperCam’s 535- to 853-nm range includes an intensifier which can amplify weak optical signals and therefore makes visible-wavelength auroral emissions detectable above the dark noise from charge-coupled device (CCD) thermal electrons. SuperCam covers several hypothesized auroral lines, such as the forbidden atomic oxygen transitions at 557.7 and 630 nm. However, the red (630 nm) line is estimated to be roughly 20 times fainter than the green (557.7 nm) line (*37*) and consequently more difficult to detect.

The SuperCam aurora observations consist of sets of 75 spectra each with 0.75-s integration time, for which means and medians but not individual spectra are downlinked. The complete observation for the data presented here are four sets of spectra: a “dark” set, followed by two illuminated sets, followed by a final dark set, with a total duration of 5 min. Dark sets are those which have the intensifier switched off to measure the detector background.

To estimate uncertainties, we perturb the best fit in-band emission line spectrum, which contains 32 data points surrounding the theoretically expected line center, by all possible sets of 32 adjacent residual data points in their original order drawn from the full set of residuals, repeating the line fit with each perturbation and thereby building up a probability distribution of fit results. The full set of residuals contains the 266 data points of residuals generated by the continuum plus emission line fit. Maintaining perturbing data points in their original order and adjacency ensures that the effects of autocorrelations in instrument noise are taken into account in the probability distribution.

#### Perseverance Mastcam-Z

Mastcam-Z is a pair of multispectral, stereoscopic imaging cameras capable of providing broadband and narrowband color images and direct solar images using neutral density filters. It has a selectable field of view (FOV) ranging from 6.2° × 4.6° to 25.6° × 19.2° (*53*, *54*). Each camera has a CCD detector with 1648 × 1200 photoactive pixels and Bayer pattern RGB (red, green and blue) microfilters. Bayer patterns have 4-pixel unit cells, each with two green (544 ± 42 nm) pixels, one red (630 ± 43 nm), and one blue (480 ± 46 nm). This arrangement of unit cells is noteworthy for the processing strategy described in the “Sky brightness” section. The Mastcam-Z aurora observation included four stereo pairs taken above the western horizon using 120-s exposures, spanning roughly 9 min in total duration, immediately after the SuperCam observation. The first and last were acquired with solar filters (10^5^ and 10^6^ neutral density for the right and left cameras, respectively) to serve as contemporaneous dark measurements, while the middle two were taken with clear filters. Mastcam-Z was at 26-mm zoom, giving a FOV of 25.6° × 19.2° and using the full resolution of 1608 × 1200 pixels plus 40 virtual dark columns.

### Mars Express memory errors

To investigate whether SEPs affected Mars close to the time of observation, we used an error detection and correction (EDAC) counter from the Mars Express (MEx) orbiter, which was made available in near real time. EDACs cumulatively count bit flips in spacecraft memories (*55*). Bit flips can be caused by the impact of charged energetic particles (solar wind particles with energies < 10 keV do not trigger an EDAC count), thus the solar cycle modulation of galactic cosmic rays is discernible in EDAC counters as a sinusoidal curve (*56*), while SEP events manifest as sudden increases (*57*, *58*). The necessary energy required to increase the EDAC counter depends on the spacecraft structure and material and will therefore differ from spacecraft to spacecraft (*59*). Sánchez-Cano *et al.* (*57*) found that protons with energies > 20 MeV are required to trigger an EDAC increment on MEx, which correspond to the proton energies found in (*33*) to contribute to the diffuse aurora.

### MAVEN SEP

To further study the energetic particles impacting Mars during the rover observation, the SEP instrument on the MAVEN satellite was used. The MAVEN/SEP instrument measures the energy spectrum and angular distribution of solar energetic electrons and ions and consists of two sensors mounted on the corners of the spacecraft, each with a FOV of by 42° × 31°. Each MAVEN/SEP sensor is a double-ended telescope that contains a solid-state detector that measures fluxes of electrons from 20 to 1000 keV and ions (mostly protons) from 20 to 6000 keV (*60*), thus covering the range of electron energies modeled to reproduce the vertical auroral emission profiles observed by the MAVEN Imaging Ultraviolet Spectrograph (*14*, *21*).

## RESULTS

### The solar storm

The analysis of the solar activity observations and the WSA-ENLIL + cone simulation results that is relevant to the Mars aurora activity reported here was performed by the NASA M2M Space Weather Analysis Office and the CCMC, respectively, and can be found at the DONKI simulation page and the web links within (https://kauai.ccmc.gsfc.nasa.gov/DONKI/view/WSA-ENLIL/29602/1). Here, we briefly provide some of the analysis details and simulation results for space weather context.

On 15 March 2024, a long-lasting C4.9 flare with an accompanying CME erupted from Active Region 13599. The CME was initially observed from the SOHO LASCO C2 coronagraph at 02:10 UTC, while the flare was observed by GOES to peak at 03:57 UTC. On the basis of initial CME observations, the ICME was parametrized with the cone tool to have an initial velocity of 1121 km/s. The modeled arrival time of the ICME leading edge was estimated to reach Mars on 18 March at 00:51 UTC, ±7 hours, with the ion density peak estimated to occur around 04:00 UTC. The modeled solar wind plasma and magnetic field values substantially exceeded the solar storm selection criteria described in the “Solar storm selection” section, as shown in Fig. 1.

**Fig. 2.**
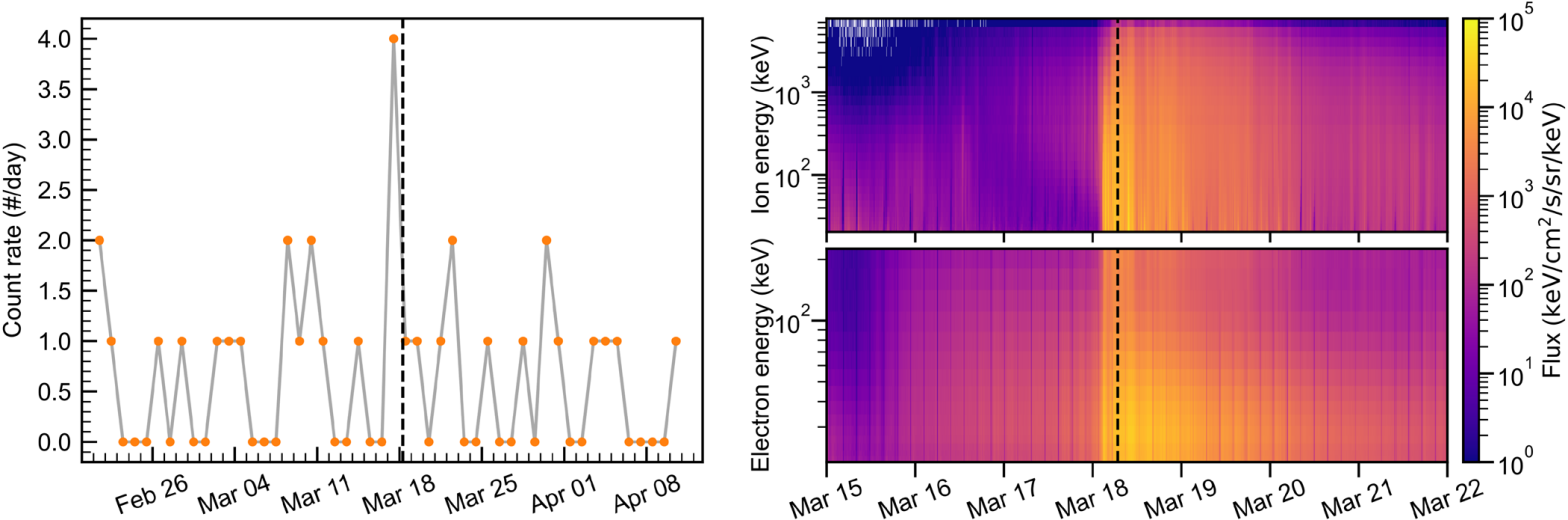
Energetic solar particles affecting Mars during the 18 March event. **Left**: MEx memory error time series. The orange dotted line represents the number of errors per day. **Right**: MAVEN/SEP electron and ion flux timeline. The color bars represent ion (top) and electron (bottom) fluxes. For all figures, the black dashed line indicates the time of the rover observations.

Figure 2 shows the impact of energetic particles at Mars. There was no increase in MEx errors or MAVEN/SEP flux immediately after 15 March, indicating that few SEPs affected Mars as a result of the flare. MEx registered an increase in errors late on 17 March, about 6 hours ahead of the MAVEN/SEP flux increase. The difference in peak times between the EDAC and MAVEN/SEP could be explained by EDACs likely being triggered by higher-energy particles (mega–electron volts), reaching Mars ahead of the bulk of the ICME which consists of less the energetic particles detected by MAVEN (kilo–electron volts). The forecast ion density peak corresponds very well with the MAVEN/SEP flux increase. MAVEN/SEP shows a simultaneous broadband increase in SEP electron and ion, similar to the SEP signatures of previous ICMEs affecting Mars (*61*).

### The rover observations

The rover activity was initiated on 18 March (mission sol 1094), with the instruments switching on at 06:30 UTC, corresponding to 00:15 local mean solar time (LMST). Diffuse aurora is assumed to be spatially uniform, and the intensity recorded at the surface is influenced by viewing elevation angle and aerosol absorption. The observations presented here were made in the Northern Hemisphere early fall at *L*_s_ = 219°, during Mars’ dusty season. Because of recent regional dust storm activity, visible-band vertical-column dust opacity (τ) was 1.1, slightly higher than seasonal normal, as measured by routine direct imaging of the Sun during the day (*62*). On the basis of preliminary assessments of stellar photometry and aerosol-scattered starlight, the true nighttime opacity may have been notably higher.

SuperCam was pointed at 20° elevation angle, to maximize the received auroral intensity. The aurora is expected to be weak and spatially uniform, so by pointing the spectrometer at a certain elevation angle, we intersect a longer path length through the emitting layer compared to if the instrument was pointed at zenith. The optimal angle depends on the dust loading and the altitude of the emitting layer. Mastcam-Z was pointed at a 15.5° elevation angle to capture the local topography at the bottom edge of the image as a visual contrast to the sky illumination. The center azimuth angle for both instruments was 252° relative to local north.

An illuminated Phobos was in the sky during the observation, which led to an increase in sky brightness. To better characterize the background night sky with no auroral emission, a follow-up observation was conducted on sol 1107 with identical sky pointing, Phobos at a similar position, comparable dust loading, and additional images acquired while Phobos was fully eclipsed.

#### Spectral line identification

The SuperCam spectra were acquired over a period of 5 min beginning at 00:32 LMST (06:46 UTC). The data are processed as follows, starting from engineering data records stored in NASA’s Planetary Data System (PDS): (i) take the median at each pixel position for each acquired 75 spectra set yielding one spectrum for each set; (ii) subtract the nearest dark spectrum from each illuminated spectrum; (iii) take the mean of the illuminated sets; (iv) perform a 3-pixel wide median filter to reduce signal spikes.

We use the same instrument response function as is used to calibrate the PDS SuperCam calibrated data records. This PDS instrument response function is as reported in (*63*) but with an additional scaling factor of 1.25 that applied when detector temperatures are below 0°C. This scaling factor is based on prelaunch laboratory testing of the SuperCam body unit (*52*). We use a wavelength calibration with pixel wavelength assignment from (*63*), which used a dataset with detector temperatures nearly identical to those of the sol 1094 observation. Note that routine LIBS observations have observed no temporal or temperature-related change in wavelength calibration, consistent with expectations from (*52*).

**Fig. 3.**
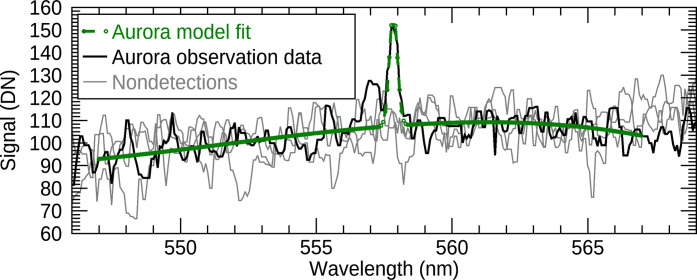
SuperCam average spectrum and best-fit model. The average of 75 × 2 spectra from the sol 1094 aurora detection observation shown in black, with the corresponding best-fit model for the continuum and the 557.7 nm emission in green. Observed spectra from nondetections on sols 790, 900, and 1107 are shown for comparison in gray, with the signal levels offset so that their averages between 559 and 566 nm are matched to that of sol 1094.

We fit a third-order polynomial continuum and then a Gaussian emission line shape to these data, with line center and integrated line intensity as the free parameters. For the width of the emission line, we use the instrumental line width of 0.35 nm FWHM from (*52*). The data and fit are shown in Fig. 3. With this fit, we obtain a line intensity of 101 ± 14 (1σ uncertainty) Rayleighs (*R*) and a line center at 557.843 ± 0.024 nm (1 *R* = 7.96 × 10^8^ photons m^−2^ sr^−1^ s^−1^). The well-known “557.7-nm” O(^1^S → ^1^D) line has a vacuum wavelength of 557.889 nm (*64*), which is within 2σ of what we obtain from the observation. The vacuum wavelength is the reference for the SuperCam wavelength calibration.

**Fig. 4.**
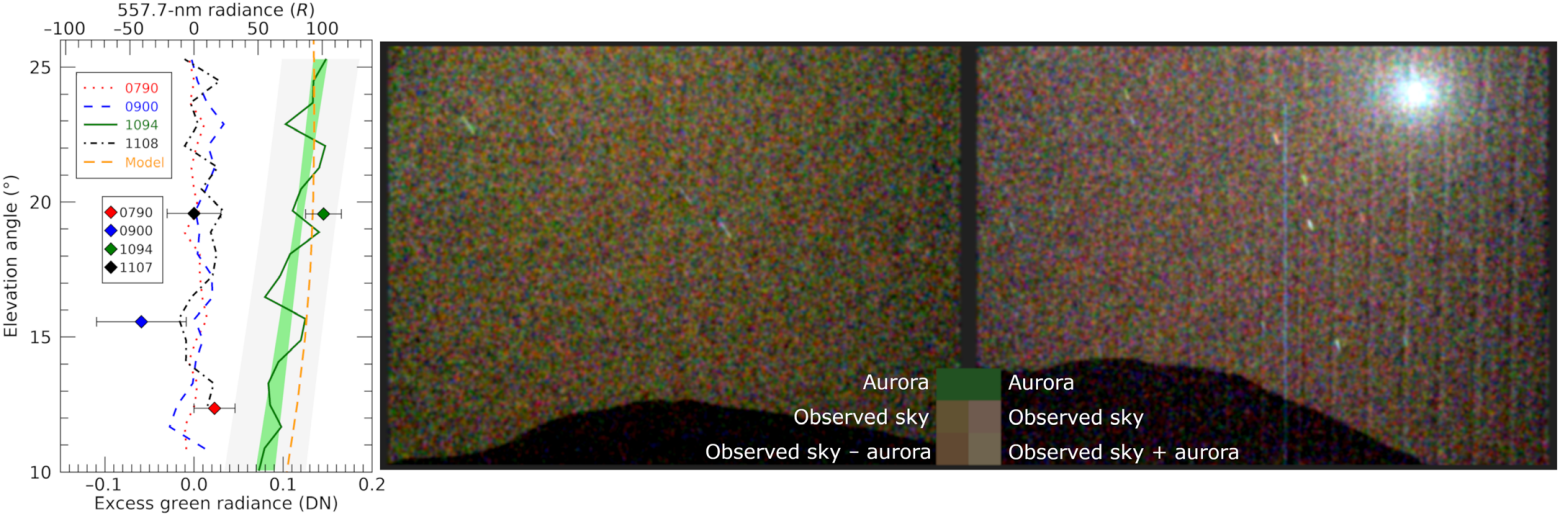
Evidence of excess green signal in the Martian sky on March 18th. Profiles of excess green signal for all four aurora detection attempts (**left**), and images of the martian sky with aurora on sol 1094 (**middle**) and on a reference sol without aurora (**right**). Both images, which have identical color stretches, aerosol-scattered Phobos-light removal, and 2-pixel Gaussian smoothing, show the martian night sky contrasted against nearby topography seen at the bottom edge. See the Supplementary Materials for further image processing details. In each image, there are three squares which show how different noise-free uniform signals would appear if measured by Mastcam-Z and presented in the color stretch used for the images. The top squares represent the 557.7-nm aurora with no other sky illumination. The middle squares show the observed average signal in the center of each image after Phobos signal removal. The bottom squares show the average signals in the center of the images, with the excess green aurora signal removed for the aurora image and added for the aurora-free image. Thus, the bottom squares illustrate the colors that an aurora-free sky would have had on sol 1094 for the left image and that an aurora-illuminated sky would have had on sol 1108 for the right image. Left: The average excess green signal (calculated as described in the text) in the left image as a function of elevation angle. Mastcam-Z profiles and the model are shown as lines, while the SuperCam radiance measurements are indicated by diamonds. The colors represent different mission sols. Only sol 1094 (solid green) yielded a positive detection. The shaded green and gray areas represent the Mastcam-Z instrumental uncertainty of the best fit and the 95% confidence interval including uncertainties due to corrections for scattered Phobos light, respectively. The orange dashed line shows an auroral line radiative transfer model calculation fit to the sol 1094 SuperCam measurement.

To validate the above results, we performed identical data processing procedures for two previous detection attempts and the follow-up observation (sols 790, 900, and 1107), all of which yielded nondetections. The signal levels and uncertainties for all four observations are plotted in Fig. 4 for comparison with the corresponding Mastcam-Z measurements.

#### Sky brightness

Mastcam-Z observations started at 00:43 LMST (06:57 UTC). Analysis of the clear filter images began with removing the dark current determined from the before-and-after dark images, where detector self-heating resulted in a cold and warm measurement. Then, all pixels of ≥5σ from the mean were masked and discarded from further consideration; these were largely radiation strikes but also included pixels with variable dark current. The remaining pixels were divided by an instrumental flat field and binned spatially to increase precision at the expense of resolution.

Phobos was fully illuminated during the observation, and scattering of its light by aerosols contributed to 0.6 to 1.9 digital numbers (DN) of the signal. The aerosol-scattered Phobos light was red-enhanced, causing the overall night sky color, at that time, to be very similar to a much less bright daytime martian sky. The Mastcam-Z signal from aerosol-scattered starlight (after excluding Phobos contribution) was found using previous dark sky images (e.g., sol 900) and was estimated to be ∼0.5 DN or ∼1.2 × 10^−6^ Wm^−2^ μm^−1^ sr^−1^ [table 7 of (*54*)] across the visible spectrum. This value is presumably dependent on aerosol opacity, especially at shorter wavelengths where aerosol dust has substantial absorption. The scattered starlight has a similar signal in each color channel (thus gray in color). Any residual did not contribute to the excess green signal (positive or negative) and was therefore not removed but might be of interest for future studies of nighttime atmospheric phenomena.

To create an image of the aurora-illuminated martian night sky with the Phobos contribution removed, a Phobos illumination model was made with the discrete-ordinate-method radiative transfer model (*65*) using Hapke parameters from (*66*) and the shape model from (*67*). The resulting irradiance was verified against a Phobos image from sol 319. The model was calculated and sampled at each pixel and then averaged in the same way as the observations. Corrected versions of the images were calculated by subtracting the modeled Phobos light. The two corrected pairs were then averaged, and the result can be seen in the left of Fig. 4. The corrected image includes aurora, scattered starlight, and any residual. The diffuse aurora covers the entire FOV of Mastcam-Z and causes the green-yellow hue of the image. There is no evidence for spatial nonuniformity of the aurora, and the brightness changes only with a viewing angle. With an integration time of 2 min, any temporal variability on shorter timescales is not detectable but cannot be ruled out on the basis of this observation. Deimos appears by coincidence in the upper right of aurora-free image. Because of the long exposure time, two stars create visible trails in the image.

To isolate the excess green signal and display it as a function of elevation angle, the RGB Bayer pattern array was separated into four color channels: two for green and one each for red and blue. A weighted average of the red and blue channels was created, which was multiplied by 1.05 to account for the higher sensitivity of the green channel (*54*). The amount of excess green light was determined by the difference between the green and the average of the red and blue channels, effectively removing white starlight and other residual white light. The unmasked pixels were then averaged across detector regions. Consistency along brightness profiles from all four images demonstrated that instrumental measurement uncertainty was <0.05 DN. Profiles from the first and second left and right images were averaged, creating the emission profile in the left of Fig. 4. The intensity is higher at larger elevation angles, where the path length through the dusty lower atmosphere from a high-altitude source is shorter.

With Phobos light removed and starlight observed to be spectrally uniform, there is no other likely source for an excess green signal, and so we attribute it to the atomic oxygen O(^1^S → ^1^D) green 557.7-nm auroral emission. We therefore map the excess green DN signal to Rayleighs using the Mastcam-Z quantum efficiency at this wavelength together with the Mastcam-Z optical characteristics [c.f. figure 20 and table 1 of (*54*), respectively]. At a 20° elevation angle, the excess green signal is 0.119 ± 0.004 DN (1σ) or 82 ± 2 *R* with instrumental uncertainties only. The uncertainty contributed by the Phobos light model is about 0.02 DN or 14 R (1σ).

Two other nights with nondetections were processed similarly; sol 900 was moonless, while sol 790 included Phobos light. In these cases, and on sol 1107, the excess green was <0.01 DN as seen in Fig. 4, and the RMS residual in the elevation profiles was <0.02 DN.

MEDA RDS did not detect aurora on 18 March 2024. Assuming the aurora was spatially uniform, that the UV and visible lines had the same relative intensities as the strong event of September 2017 reported in (*21*), and using the 16.5 transitional probability factor (*21*), we estimate an integrated UV brightness of 4 × 10^−8^ Wm^−2^ in the RDS sensor covering the 250- to 319-nm range for the September 2017 event in (*21*), which is two to three times below the sensitivity threshold for the UV RDS sensor. The 18 March 2024 event was an order of magnitude weaker than the September 2017 event (see Discussion), so no MEDA RDS detection would be expected for either event.

To put the observed sky brightness in context, we made preliminary aurora brightness calculations using the multiple-scattering pseudo-spherical radiative transfer code of (*68*). We replaced the 1270-nm O_2_ (^1^∆_g_) volume emission from (*68*) with the 557.7-nm line volume emission with a vertical profile shaped to match the relative brightness by altitude of the 297.2-nm emission observed for the September 2017 event (*21*) and used aerosol column opacity as observed for mission sol 1094 (i.e., τ = 1.1). The original version of this code, developed by Smith *et al.* (*69*), was designed for any wavelength from 300 to 3900 nm; therefore, no other changes to the code used by Clancy *et al.* (*68*) were required. The results proved to be insensitive to the aerosol vertical profile and insensitive to aerosol properties other than total column opacity. Using this code, we calculate the brightness–versus–elevation angle profile in Fig. 4 (orange dashed line) and estimate that the observed 101 *R* intensity would have been 2.1 kR if viewed from orbit on the limb. Applying the transitional probability factor of 16.5 from (*35*), we estimate that the O(^1^S → ^3^P) line at 297.2 nm would produce a signal of 130 *R* on the limb, 10 times fainter than the September 2017 event (*21*) observed by MAVEN.

The radiative transfer model also indicates that, as expected, the same aurora emission rate would yield substantially brighter aurora as observed from the surface if atmospheric conditions were clear. For example, with τ = 0.3 (near the minimum value observed by Perseverance), the 101 *R* would have been approximately 200 *R* at the brightest elevation angle (10° due to the low opacity), which is just below the threshold of what may be perceptible to the human eye [Lilensten *et al.* (*37*) give a threshold of 250 *R*]. If the solar event was as intense as the September 2017 event, the green line emission would lead to a sky brightness of about 2 kR in τ = 0.3 clear conditions or 1 kR with conditions as dusty as during the current observation. Both situations would be visible to the human eye as a glow above the horizon.

## DISCUSSION

On 15 March 2024, a minor solar flare was followed by a larger ICME, modeled by NASA CCMC to produce a SEP event at Mars on 18 March. A space weather alert notification was issued, which provided enough lead time to schedule SuperCam and Mastcam-Z auroral observations in response to the expected SEP impact. SuperCam identified the 557.7-nm O(^1^S → ^1^D) green emission line with an intensity of 101 ± 14 *R*. An excess green signal in the Mastcam-Z image was attributed to the same emission.

We discard the possibility of dust or clouds causing the observed emission line, as aerosol scattered light would lead to a broad-band feature. We also find it unlikely that this is a nightglow phenomenon resulting from dayside ionization, as no similar signal was found in the previous detection attempts or in the follow-up observation. In addition, green martian nightglow manifests through the O_2_ Herzberg II emission (*70*) and the much weaker Chamberlain system, with closest peaks at 552 and 558 nm, respectively (*71*). As there is no other likely source of green light, the 557.7-nm emission line is attributed to an auroral event.

The 0.12 ± 0.02 DN (1σ) at 20° elevation angle observed by Mastcam-Z (the same angle as SuperCam’s observation) equates to 82 ± 14 *R*. This is fully consistent with the SuperCam measurement, as the cross-calibration is not expected to be perfect between the two instruments. The two measurements were also conducted some minutes apart, so temporal variations may also have affected the difference in retrieved brightness. The apparent elevation angle gradient retrieved from the Mastcam-Z image matches radiative transfer model expectations for a horizontally uniform source at ionospheric altitudes.

Considering that C-class flares are unlikely to produce SEPs (*72*) and that the MAVEN/SEP and MEx EDAC peak coincided with the estimated arrival of the ICME, we conclude that the observed aurora was mainly induced by particles accelerated by the ICME-driven shock. To determine the relative contributions to the emission from kilo–electron volt electrons and mega–electron volt protons, further investigation is required. While the brightness of this event was dimmed by dust, events under better viewing conditions or more intense particle precipitation might be above the threshold for human vision and visible to future astronauts.

Studying auroras at multiple wavelengths is a powerful tool, as the ratio between emissions can be used to constrain the incoming particle energy distribution (*1*, *2*), and provides insight into the atmospheric composition and pressure. In particular, the ratio of green to red intensity can be used as an indicator of the incoming particle energy distribution; higher energies favor the O(^1^S → ^1^D) 557.7-nm green line over the O(^1^D → ^3^P) 630- to 636-nm red doublet, as higher-energy particles penetrate deeper into the atmosphere where the emission from the O(^1^D → ^3^P) transition is suppressed by collisional quenching. During this particular event, no other emission lines were detected by SuperCam, but an upper limit for the 630-nm red line was derived to be ∼50 *R*, although its expected brightness is a factor of 20 lower than the 557.7-nm line.

This detection confirms the prediction from UV observations that emissions also occur at visible wavelengths [e.g., (*4*, *73*)] and demonstrates that auroral forecasting is possible. The presence of visible aurora opens a new avenue for the study of space weather events at Mars; visible imagers and spectrometers can be simpler and lower cost than the UV instrumentation used to date for the study of martian aurora, especially if using wide-angle optics with narrow-band filters tuned to specific visible auroral emission lines, such as the Mars Aurora and Dust Camera included in the proposed M-MATISSE mission (*74*).

The detection of visible-wavelength aurora from the surface also enables the potential future investigation of temporal auroral variability and spatial structures, observations not easily obtained from orbit with current instrumentation. Spatial and/or temporal variations in the emission intensity will reveal the degree of rapid dynamics in the magnetotail. Follow-up studies will include adapted observation strategies to attempt to capture any variability, as well as in-depth analysis of concurrent plasma and SEP measurements from orbit and other surface assets, and comparisons with data acquired during additional space weather events which followed this first detection.

## Supplementary Materials

### This PDF file includes:

Supplementary Text Figs. S1 and S2 References
