# Supplementary material for: Detection of visible-wavelength aurora on Mars
Source: Sci Adv. 2025 May 14;11(20):eads1563. doi: 10.1126/sciadv.ads1563 (PMC12077521; doi:10.1126/sciadv.ads1563)
Supplement: Supplementary file 1 — Supplementary Text Figs. S1 and S2 References [file sciadv.ads1563_sm.pdf]

Science Advances
AAAS

Supplementary Materials for

Detection of visible-wavelength aurora on Mars

Elise W. Knutsen *et al.*

Corresponding author: Elise W. Knutsen, elisewkn@uio.no

Sci. Adv. **11**, eads1563 (2025)
DOI: 10.1126/sciadv.ads1563

This PDF file includes:

Supplementary Text
Figs. S1 and S2
References

Supplementary materials

1. Phobos light contribution to the Martian night sky

We used radiative transfer (RT) modeling to isolate the auroral signal in images that included scattered light from Phobos. We considered the sol-1094 images to include three light sources. (1) Scattered light from Phobos—this is sunlight scattered off the surface of Phobos and scattered again in the sky, as Phobos was far from the field of view. (2) Other broadband light, primarily starlight. (3) The auroral line, for which the green microfilter was ~20x more sensitive than red or blue. Among these, (1) was the only light source with a strong color component. Due to the ~0.6-1.9 DN Phobos signal being much larger than the auroral signal (~0.1 DN) and starlight (~0.5 DN), we modeled it for removal from the observations.

Rather than model starlight (both scattered light from all stars and direct light from unresolved stars), we used the excess green metric to remove it. The difference between the green signal and the weighted average of the red and blue signals is sensitive to the auroral line but not to any flat or linear spectrum (blue slope to gray to red slope). Based on previous nights with no detected aurora and no Phobos signal, starlight varies with local conditions but is spectrally flat within the Mastcam-Z bandpass. In any case, the excess green metric shows no auroral detection, which agrees with SuperCam.

The first step in the RT modeling was to determine the incident flux. Hapke parameters from [66] and the shape model from [67] were used with geometric parameters determined from the JPL ephemeris. This intermediate result was verified against a sol-319 image of Phobos with Mastcam-Z using the same filter as the auroral observations.

The second step was to use DISORT [65] to determine radiance from the sky. The dust model [75] used a radius and variance of 1.6 microns and 0.3, respectively, with indices of refraction from [76]. Only dust and Rayleigh scattering were considered, with the dust modeled as well-mixed with the gas atmosphere below 100 km altitude (so the extinction is almost entirely within 0-40 km altitude given the 10.5-km scale height) and the total column dust optical depth taken from daytime solar extinction measurements [60]. DISORT was initialized with 160 streams and 256 moments and set to output a grid of radiances for scattering azimuth of 0 to 180 degrees in 0.25-degree steps and 0 to 80 zenith angles in 0.25-degree steps. Calculations were made for band centers at 480, 544, and 630 nm. Using solar fluxes, the result was validated using daytime sky images with the Sun near the same elevation as Phobos for the night images. For angles below 10-deg elevation, the radiance at 10-deg was used for cosmetic purposes, but such angles were not included in the analysis.

2. Aurora emission model

The SuperCam spectral residuals are used to estimate the uncertainties, as described in the last paragraph of section 2.2.1 in the main text.

Figure S2 shows the full SuperCam spectrum, where the 557.7 nm auroral line is distinguishable. None of the airglow emission lines of O_2, the Herzberg II (552 nm, 594 nm) or the much weaker Chamberlain bands (558 nm, 604 nm), are visible.

3. Additional discussion of Figure 4 images processing and remaining image artifacts.

After the data for the graph in Figure 4 was extracted, we performed some additional image processing to reduce noise and remove saturated pixels in the images as they are shown in the center and right of Figure 4. The additional processing is as follows: We first smooth with a sigma = 2 pixel gaussian kernel to reduce noise. Invalid pixels are ignored when calculating the smoothed result. Invalid pixels include cosmic ray hits, hot pixels, and pixels that are considered "saturated" because their signal levels exceed the linear range of the detector. In addition to reducing noise for all pixels, the smoothing replaces invalid pixels with the smoothed result calculated from neighboring pixels. Next, because the invalid-pixel replacement does not work well for large clusters of invalid pixels, we set the 58 pixels that are saturated by Deimos (which appears in the upper right of the right-hand image) equal to the maximum value in the smoothed image adjacent to Deimos. Note that the maximum of the color stretch we use in displaying the images is significantly less than the sky brightness immediately adjacent to Deimos, so both Deimos and a small area of sky surrounding Deimos appear with a maxed-out color stretch.

The vertical stripes that remain in the displayed images are caused by residuals in the detector background subtraction, likely caused by temporal variability in the detector electronics – they are apparent only because illumination conditions are roughly 6 orders of magnitude fainter than Mastcam-Z system was designed for.

One vertical white line in the right-hand image is known to be caused instead by a residual from hot pixel removal – the hot pixel itself has been flagged as invalid and removed, but the one pixel has caused a faint contamination signal in other pixels in the same detector column during detector read-out. Once again, this faint contamination is only apparent because of the very low illumination conditions.

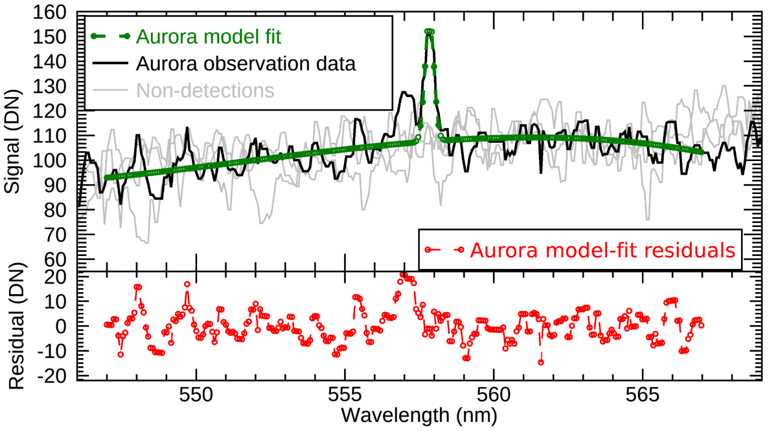

Figure S1. SuperCam average spectrum and residual after fitting aurora model. Top panel: the average of 75×2 spectra from the sol 1094 aurora detection observation shown in black, with the corresponding best-fit model for the continuum and the 557.7 nm emission in green. Observed spectra from non-detections on sols 790, 900 and 1107 are shown for comparison, with the signal levels offset so that their averages between 559-588 nm are matched to that of sol 1094. Bottom panel: Residual spectrum from aurora observation is shown in red.

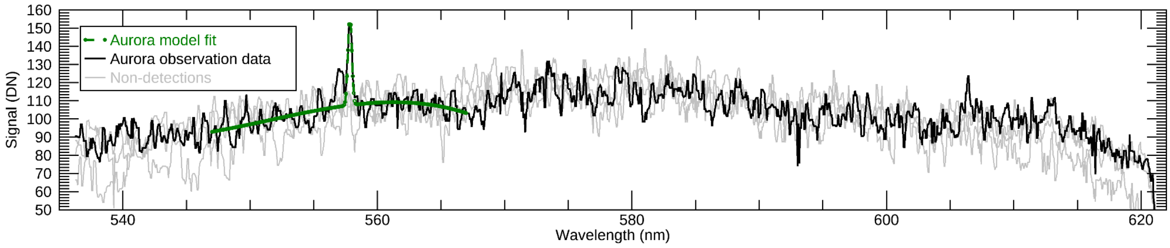

Figure S2: Full SuperCam spectrum. Data from the full wavelength range of SuperCam is shown, with the aurora model fitted to part of the spectrum where the green 557.7 nm oxygen line was expected.

REFERENCES AND NOTES

1. M. H. Rees, D. Luckey, Auroral electron energy derived from ratio of spectroscopic emissions 1. Model computations. *J. Geophys. Res.* **79**, 5181–5186 (1974).

2. L. Soret, B. Hubert, J. C. Gérard, S. Jain, K. Chirakkil, R. Lillis, J. Deighan Quantifying the electron energy of Mars aurorae through the oxygen emission brightness ratio at 130.4 and 135.6 nm. *J. Geophys. Res. Planets* **129**, e2023JE008214 (2024**)**.

3. L. Soret, J. C. Gérard, L. Libert, V. I. Shematovich, D. V. Bisikalo, A. Stiepen, J.-L. Bertaux SPICAM observations and modeling of Mars aurorae. *Icarus* **264**, 398–406 (2016).

4. F. González-Galindo, J.-C. Gérard, L. Soret, J.-Y. Chaufray, A. Fedorova, M. Holmstrom, F. Lefèvre, M. Á. López-Valverde, F. Montmessin Airglow and aurora in the martian atmosphere: Contributions by the Mars Express and ExoMars TGO missions. *Space Sci. Rev.* **220**, 42 (2024).

5. L. J. Paxton, Y. Zhang, H. Kil, R. K. Schaefer, "Exploring the upper atmosphere: Using optical remote sensing" in *Geophysical Monograph Series*, W. Wang, Y. Zhang, L. J. Paxton, Eds. (Wiley, 2021), pp. 487–522.

6. M. H. Acuña, J. E. Connerney, N. F. Ness, R. P. Lin, D. Mitchell, C. W. Carlson, J. McFadden, K. A. Anderson, H. Reme, C. Mazelle, D. Vignes, P. Wasilewski, P. Cloutier, Global distribution of crustal magnetization discovered by the Mars global surveyor MAG/ER experiment. *Science* **284**, 790–793 (1999).

7. D. A. Brain, F. Bagenal, M. H. Acuña, J. E. P. Connerney Martian magnetic morphology: Contributions from the solar wind and crust. *J. Geophys. Res. Space Phys.* **108**, 2002JA009482 (2003).

8. D. L. Mitchell, R. J. Lillis, R. P. Lin, J. E. P. Connerney, M. H. Acuña, A global map of Mars' crustal magnetic field based on electron reflectometry. *J. Geophys. Res.*, **112**, E01002 (2007).

9. B. Sánchez-Cano, M. Lester, D. J. Andrews, H. Opgenoorth, R. Lillis, F. Leblanc, C. M. Fowler, X. Fang, O. Vaisberg, M. Mayyasi, M. Holmberg, J. Guo, M. Hamrin, C. Mazelle, K. Peter, M. Pätzold, K. Stergiopoulou, C. Goetz, V. N. Ermakov, S. Shuvalov, J. A. Wild, P. L. Blelly, M. Mendillo, C.

Bertucci, M. Cartacci, R. Orosei, F. Chu, A. J. Kopf, Z. Girazian, M. T. Roman, Mars' plasma system. Scientific potential of coordinated multipoint missions: "The next generation". *Exp. Astron.* **54**, 641–676 (2022).

10. R. J. Lillis, J. Deighan, K. Chirakkil, S. Jain, M. Fillingim, M. Chaffin, G. Holsclaw, R. Susarla, D. Brain, H. al Matroushi, F. Lootah, H. al Mazmi, Y. Dong, N. Schneider, A. Azari, R. Ramstad, M. Nauth, Y. Ma, J. Halekas, J. Espley, S. Curry Sinuous aurora at Mars: A link to the tail current sheet? *J. Geophys. Res. Space Phys.* **129**, e2024JA032477 (2024).

11. Y. Harada, J. S. Halekas, J. P. McFadden, J. Espley, G. A. DiBraccio, D. L. Mitchell, C. Mazelle, D. A. Brain, L. Andersson, Y. J. Ma, D. E. Larson, S. Xu, T. Hara, S. Ruhunusiri, R. Livi, B. M. Jakosky Survey of magnetic reconnection signatures in the martian magnetotail with MAVEN: TAIL RECONNECTION AT MARS. *J. Geophys. Res. Space Phys.*, **122**, 5114–5131 (2017).

12. J.-L.Bertaux, F. Leblanc, O. Witasse, E. Quemerais, J. Lilensten, S. A. Stern, B. Sandel, O. Korablev Discovery of an aurora on Mars. *Nature* **435**, 790–794 (2005).

13. K. Chirakkil, R. J. Lillis, J. Deighan, M. S. Chaffin, S. K. Jain, D. A. Brain, M. O. Fillingim, R. Susarla, G. Holsclaw, X. Fang, N. M. Schneider, H. A. Mazmi, H. A. Matroushi, M. Gacesa, N. El-Kork, E. Thiemann, J. S. Halekas, EMM EMUS observations of FUV aurora on Mars: Dependence on magnetic topology, local time, and season. *J. Geophys. Res. Planets* **129**, e2024JE008336 (2024).

14. N. M. Schneider, J. I. Deighan, S. K. Jain, A. Stiepen, A. I. F. Stewart, D. Larson, D. L. Mitchell, C. Mazelle, C. O. Lee, R. J. Lillis, J. S. Evans, D. Brain, M. H. Stevens, W. E. McClintock, M. S. Chaffin, M. Crismani, G. M. Holsclaw, F. Lefevre, D. Y. Lo, J. T. Clarke, F. Montmessin, B. M. Jakosky Discovery of diffuse aurora on Mars. *Science,* **350**, aad0313 (2015).

15. J. Deighan, S. K. Jain, M. S. Chaffin, X. Fang, J. S. Halekas, J. T. Clarke, N. M. Schneider, A. I. F. Stewart, J. Y. Chaufray, J. S. Evans, M. H. Stevens, M. Mayyasi, A. Stiepen, M. Crismani, W. E. McClintock, G. M. Holsclaw, D. Y. Lo, F. Montmessin, F. Lefèvre, B. M. Jakosky Discovery of a proton aurora at Mars. *Nat. Astron.* **2**, 802–807 (2018).

16. R. J. Lillis, J. Deighan, D. Brain, M. Fillingim, S. Jain, M. Chaffin, S. England, G. Holsclaw, K. Chirakkil, H. A. Matroushi, F. Lootah, H. A. Mazmi, E. Thiemann, F. Eparvier, N. Schneider, S. Curry, First synoptic images of FUV discrete aurora and discovery of sinuous aurora at Mars by EMM EMUS. *Geophys. Res. Lett.* **49**, e2022GL099820 (2022).

17. D. A. Brain, J. S. Halekas, L. M. Peticolas, R. P. Lin, J. G. Luhmann, D. L. Mitchell, G. T. Delory, S. W. Bougher, M. H. Acuña, H. Rème On the origin of aurorae on Mars. *Geophys. Res. Lett.* **33**, 2005GL024782 (2006).

18. L. Soret, J.-C. Gérard, N. Schneider, S. Jain, Z. Milby, B. Ritter, B. Hubert, T. Weber Discrete aurora on Mars: Spectral properties, vertical profiles, and electron energies. *J. Geophys. Res. Space Phys.* **126**, e2021JA029495 (2021).

19. Y. Harada, Y. Fujiwara, R. J. Lillis, J. Deighan, H. Nakagawa, B. Sánchez-Cano, M. Lester, Y. Futaana, M. Holmström, R. A. Frahm Discrete aurora and the nightside ionosphere of Mars: An EMM–MEX conjunction of FUV imaging, ionospheric radar sounding, and suprathermal electron measurements. *Earth Planets Space* **76**, 64 (2024).

20. B. M. Jakosky, J. M. Grebowsky, J. G. Luhmann, J. Connerney, F. Eparvier, R. Ergun, J. Halekas, D. Larson, P. Mahaffy, J. McFadden, D. F. Mitchell, N. Schneider, R. Zurek, S. Bougher, D. Brain, Y. J. Ma, C. Mazelle, L. Andersson, D. Andrews, D. Baird, D. Baker, J. M. Bell, M. Benna, M. Chaffin, P. Chamberlin, Y.-Y. Chaufray, J. Clarke, G. Collinson, M. Combi, F. Crary, T. Cravens, M. Crismani, S. Curry, D. Curtis, J. Deighan, G. Delory, R. Dewey, G. DiBraccio, C. Dong, Y. Dong, P. Dunn, M. Elrod, S. England, A. Eriksson, J. Espley, S. Evans, X. Fang, M. Fillingim, K. Fortier, C. M. Fowler, J. Fox, H. Gröller, S. Guzewich, T. Hara, Y. Harada, G. Holsclaw, S. K. Jain, R. Jolitz, F. Leblanc, C. O. Lee, Y. Lee, F. Lefevre, R. Lillis, R. Livi, D. Lo, M. Mayyasi, W. McClintock, T. McEnulty, R. Modolo, F. Montmessin, M. Morooka, A. Nagy, K. Olsen, W. Peterson, A. Rahmati, S. Ruhunusiri, C. T. Russell, S. Sakai, J.-A. Sauvaud, K. Seki, M. Steckiewicz, M. Stevens, A. I. F. Stewart, A. Stiepen, S. Stone, V. Tenishev, E. Thiemann, R. Tolson, D. Toublanc, M. Vogt, T. Weber, P. Withers, T. Woods, R. Yelle MAVEN observations of the response of Mars to an interplanetary coronal mass ejection. *Science* **350**, aad0210 (2015).

21. N. M. Schneider, S. K. Jain, J. Deighan, C. R. Nasr, D. A. Brain, D. Larson, R. Lillis, A. Rahmati, J. S. Halekas, C. O. Lee, M. S. Chaffin, A. Stiepen, M. Crismani, J. S. Evans, M. H. Stevens, D. Y. Lo, W. E. McClintock, A. I. F. Stewart, R. V. Yelle, J. T. Clarke, G. M. Holsclaw, F. Lefevre, F. Montmessin, B. M. Jakosky Global aurora on Mars during the September 2017 space weather event. *Geophys. Res. Lett.* **45**, 7391–7398 (2018).

22. M. Desai,J. Giacalone, Large gradual solar energetic particle events. *Living Rev. Sol. Phys.* **13**, 3 (2016).

23. D. V. Reames, Element abundances in impulsive solar energetic-particle events. *Universe* **9**, 466 (2023).

24. D. V. Reames, Particle acceleration at the Sun and in the heliosphere. *Space Sci. Rev.* **90**, 413–491 (1999).

25. M. J. Owens, R. J. Forsyth, The heliospheric magnetic field. *Living Rev. Sol. Phys* **10**, 5 (2013).

26. Y. Futaana, S. Barabash, M. Yamauchi, S. McKenna-Lawlor, R. Lundin, J. G. Luhmann, D. Brain, E. Carlsson, J.-A. Sauvaud, J. D. Winningham, R. A. Frahm, P. Wurz, M. Holmström, H. Gunell, E. Kallio, W. Baumjohann, H. Lammer, J. R. Sharber, K. C. Hsieh, H. Andersson, A. Grigoriev, K. Brinkfeldt, H. Nilsson, K. Asamura, T. L. Zhang, A. J. Coates, D. R. Linder, D. O. Kataria, C. C. Curtis, B. R. Sandel, A. Fedorov, C. Mazelle, J.-J. Thocaven, M. Grande, H. E. J. Koskinen, T. Sales, W. Schmidt, P. Riihela, J. Kozyra, N. Krupp, J. Woch, M. Fränz, E. Dubinin, S. Orsini, R. Cerulli-Irelli, A. Mura, A. Milillo, M. Maggi, E. Roelof, P. Brandt, K. Szego, J. Scherrer, P. Bochsler Mars Express and Venus Express multi-point observations of geoeffective solar flare events in December 2006. *Planet. Space Sci.*, **56**, 873–880 (2008).

27. A. Posner, R. D. Strauss, Warning time analysis from SEP simulations of a two-tier REleASE system applied to Mars exploration. *Space Weather* **18**, e2019SW002354 (2020).

28. C. O. Lee, B. M. Jakosky, J. G. Luhmann, D. A. Brain, M. L. Mays, D. M. Hassler, M. Holmström, D. E. Larson, D. L. Mitchell, C. Mazelle, J. S. Halekas Observations and impacts of the 10 September 2017

solar events at Mars: An overview and synthesis of the initial results. *Geophys. Res. Lett.* **45**, 8871–8885 (2018).

29. F. Leblanc, J. G. Luhman, R. E. Johnson, E. Chassefiere, Some expected impacts of a solar energetic particle event at Mars. *J. Geophys. Res. Space Phys.* **107**, SIA5-1–SIA5-10 (2002).

30. R. D. Jolitz, C. F. Dong, A. Rahmati, D. A. Brain, C. O. Lee, R. J. Lillis, S. M. Curry, B. M. Jakosky Test particle model predictions of SEP electron transport and precipitation at Mars. *J. Geophys. Res. Space Phys.* **126**, e2021JA029132 (2021).

31. J. S. Halekas, D. A. Brain, J. G. Luhmann, G. A. DiBraccio, S. Ruhunusiri, Y. Harada, C. M. Fowler, D. L. Mitchell, J. E. P. Connerney, J. R. Espley, C. Mazelle, B. M. Jakosky Flows, fields, and forces in the Mars-solar wind interaction. *J. Geophys. Res. Space Phys.* **122**, 11,320–11,341 (2017).

32. J. G. Luhmann, C. F. Dong, Y. J. Ma, S. M. Curry, S. Xu, C. O. Lee, T. Hara, J. Halekas, Y. Li, J. R. Gruesbeck, J. Espley, D. A. Brain, C. T. Russell, B. M. Jakosky Martian magnetic storms. *J. Geophys. Res. Space Phys.* **122**, 6185–6209 (2017).

33. Y. Nakamura, N. Terada, F. Leblanc, A. Rahmati, H. Nakagawa, S. Sakai, S. Hiruba, R. Kataoka, K. Murase, Modeling of diffuse auroral emission at Mars: Contribution of MeV protons. *J. Geophys. Res. Space Phys.* **127**, e2021JA029914 (2022).

34. J.-C. Gérard, L. Soret, V. I. Shematovich, D. V. Bisikalo, S. W. Bougher The Mars diffuse aurora: A model of ultraviolet and visible emissions. *Icarus* **288**, 284–294 (2017).

35. J.-C.Gérard, S. Aoki, Y. Willame, L. Gkouvelis, C. Depiesse, I. R. Thomas, B. Ristic, A. C. Vandaele, F. Daerden, B. Hubert, J. Mason, M. R. Patel, J. J. López-Moreno, G. Bellucci, M. A. López-Valverde, B. Beeckman Detection of green line emission in the dayside atmosphere of Mars from NOMAD-TGO observations. *Nat. Astron.* **4**, 1049–1052 (2020).

36. L. Soret, J. C. Gérard, S. Aoki, L. Gkouvelis, I. R. Thomas, B. Ristic, B. Hubert, Y. Willame, C. Depiesse, A. C. Vandaele, M. R. Patel, J. P. Mason, F. Daerden, J. J. López-Moreno, G. Bellucci The Mars oxygen visible dayglow: A martian year of NOMAD/UVIS observations. *J. Geophys. Res. Planets* **127**, e2022JE007220 (2022).

37. J. Lilensten, D. Bernard, M. Barthélémy, G. Gronoff, C. Simon Wedlund, A. Opitz Prediction of blue, red and green aurorae at Mars. *Planet. Space Sci.*, **115**, 48–56 (2015).

38. T. G. Slanger, P. C. Cosby, D. L. Huestis, T. A. Bida Discovery of the atomic oxygen green line in the Venus night airglow. *Science* **291**, 463–465 (2001).

39. C. L. Gray, N. J. Chanover, T. G. Slanger, K. Molaverdikhani The effect of solar flares, coronal mass ejections, and solar wind streams on Venus' 5577Å oxygen green line. *Icarus* **233**, 342–347 (2014).

40. M. E. Cuesta, D. J. McComas, L. Y. Khoo, R. Bandyopadhyay, T. Sharma, M. M. Shen, J. S. Rankin, A. T. Cummings, J. R. Szalay, C. M. S. Cohen, N. A. Schwadron, R. Chhiber, F. Pecora, W. H. Matthaeus, R. A. Leske, M. L. Stevens Correlation of coronal mass ejection shock temperature with solar energetic particle intensity. *Astrophys. J.* **964**, 114 (2024).

41. Z. Girazian, N. M. Schneider, Z. Milby, X. Fang, J. Halekas, T. Weber, S. K. Jain, J. C. Gérard, L. Soret, J. Deighan, C. O. Lee Discrete aurora at Mars: Dependence on upstream solar wind conditions. *J. Geophys. Res. Space Phys.* **127**, e2021JA030238 (2022).

42. X. Fang, Y. Ma, N. Schneider, Z. Girazian, J. Luhmann, Z. Milby, S. Jain, Y. Dong, S. Curry, B. Jakosky, Discrete aurora on the nightside of Mars: Occurrence location and probability. *J. Geophys. Res. Space Phys.* **127**, e2021JA029716 (2022).

43. D. Odstrčil, V. J. Pizzo, Three-dimensional propagation of coronal mass ejections (CMEs) in a structured solar wind flow: 1. CME launched within the streamer belt. *J. Geophys. Res. Space Phys.* **104**, 483–492 (1999).

44. D. Odstrcil, Modeling 3-D solar wind structure. *Adv. Space Res.* **32**, 497–506 (2003).

45. C. N. Arge, J. G. Luhmann, D. Odstrcil, C. J. Schrijver, Y. Li, Stream structure and coronal sources of the solar wind during the May 12th, 1997 CME. *J. Atmos. Sol. Terr. Phys.* **66**, 1295–1309 (2004).

46. G. Millward, D. Biesecker, V. Pizzo, C. A. de Koning An operational software tool for the analysis of coronagraph images: Determining CME parameters for input into the WSA-Enlil heliospheric model. *Space Weather* **11**, 57–68 (2013).

47. R. Ramstad, S. Barabash, Y. Futaana, M. Yamauchi, H. Nilsson, M. Holmström, Mars under primordial solar wind conditions: Mars Express observations of the strongest CME detected at Mars under solar cycle #24 and its impact on atmospheric ion escape. *Geophys. Res. Lett.* **44**, 10,805–10,811 (2017).

48. K. A. Farley, K. H. Williford, K. M. Stack, R. Bhartia, A. Chen, M. de la Torre, K. Hand, Y. Goreva, C. D. K. Herd, R. Hueso, Y. Liu, J. N. Maki, G. Martinez, R. C. Moeller, A. Nelessen, C. E. Newman, D. Nunes, A. Ponce, N. Spanovich, P. A. Willis, L. W. Beegle, J. F. Bell III, A. J. Brown, S. E. Hamran, J. A. Hurowitz, S. Maurice, D. A. Paige, J. A. Rodriguez-Manfredi, M. Schulte, R. C. Wiens Mars 2020 mission overview. *Space Sci. Rev.* **216**, 142 (2020).

49. V. Apestigue, A. Gonzalo, J. J. Jiménez, J. Boland, M. Lemmon, J. R. de Mingo, E. García-Menendez, J. Rivas, J. Azcue, L. Bastide, N. Andrés-Santiuste, J. Martínez-Oter, M. González-Guerrero, A. Martin-Ortega, D. Toledo, F. J. Alvarez-Rios, F. Serrano, B. Martín-Vodopivec, J. Manzano, R. L. Heredero, I. Carrasco, S. Aparicio, Á. Carretero, D. R. Mac Donald, L. B. Moore, M. Á. Alcacera, J. A. Fernández-Viguri, I. Martín, M. Yela, M. Álvarez, P. Manzano, J. A. Martín, J. C. Del Hoyo, M. Reina, R. Urqui, J. A. Rodriguez-Manfredi, M. de la Torre Juárez, C. Hernandez, E. Cordoba, R. Leiter, A. Thompson, S. Madsen, M. D. Smith, D. Viúdez-Moreiras, A. Saiz-Lopez, A. Sánchez-Lavega, L. Gomez-Martín, G. M. Martínez, F. J. Gómez-Elvira, I. Arruego, Radiation and dust sensor for Mars environmental dynamic analyzer onboard M2020 rover. *Sensors*, **22**, 2907 (2022).

50. J. A. Rodriguez-Manfredi, M. de la Torre Juárez, A. Alonso, V. Apéstigue, I. Arruego, T. Atienza, D. Banfield, J. Boland, M. A. Carrera, L. Castañer, J. Ceballos, H. Chen-Chen, A. Cobos, P. G. Conrad, E. Cordoba, T. del Río-Gaztelurrutia, A. de Vicente-Retortillo, M. Domínguez-Pumar, S. Espejo, A. G. Fairen, A. Fernández-Palma, R. Ferrándiz, F. Ferri, E. Fischer, A. García-Manchado, M. García-Villadangos, M. Genzer, S. Giménez, J. Gómez-Elvira, F. Gómez, S. D. Guzewich, A. M. Harri, C. D. Hernández, M. Hieta, R. Hueso, I. Jaakonaho, J. J. Jiménez, V. Jiménez, A. Larman, R. Leiter, A. Lepinette, M. T. Lemmon, G. López, S. N. Madsen, T. Mäkinen, M. Marín, J. Martín-Soler, G. Martínez, A. Molina, L. Mora-Sotomayor, J. F. Moreno-Álvarez, S. Navarro, C. E. Newman, C. Ortega, M. C. Parrondo, V. Peinado, A. Peña, I. Pérez-Grande, S. Pérez-Hoyos, J. Pla-García, J. Polkko, M. Postigo, O. Prieto-Ballesteros, S. C. R. Rafkin, M. Ramos, M. I. Richardson, J. Romeral, C. Romero, K. D. Runyon, A. Saiz-Lopez, A. Sánchez-Lavega, I. Sard, J. T. Schofield, E. Sebastian, M. D. Smith, R. J. Sullivan, L. K. Tamppari, A. D. Thompson, D. Toledo, F. Torrero, J. Torres, R. Urquí, T. Velasco, D.

Viúdez-Moreiras, S. Zurita, The MEDA team A suite of environmental sensors for the Mars 2020 mission. *Space Sci. Rev.* **217**, 48 (2021).

51. S. Maurice, R. C. Wiens, P. Bernardi, P. Caïs, S. Robinson, T. Nelson, O. Gasnault, J. M. Reess, M. Deleuze, F. Rull, J. A. Manrique, S. Abbaki, R. B. Anderson, Y. André, S. M. Angel, G. Arana, T. Battault, P. Beck, K. Benzerara, S. Bernard, J. P. Berthias, O. Beyssac, M. Bonafous, B. Bousquet, M. Boutillier, A. Cadu, K. Castro, F. Chapron, B. Chide, K. Clark, E. Clavé, S. Clegg, E. Cloutis, C. Collin, E. C. Cordoba, A. Cousin, J. C. Dameury, W. D'Anna, Y. Daydou, A. Debus, L. Deflores, E. Dehouck, D. Delapp, G. de Los Santos, C. Donny, A. Doressoundiram, G. Dromart, B. Dubois, A. Dufour, M. Dupieux, M. Egan, J. Ervin, C. Fabre, A. Fau, W. Fischer, O. Forni, T. Fouchet, J. Frydenvang, S. Gauffre, M. Gauthier, V. Gharakanian, O. Gilard, I. Gontijo, R. Gonzalez, D. Granena, J. Grotzinger, R. Hassen-Khodja, M. Heim, Y. Hello, G. Hervet, O. Humeau, X. Jacob, S. Jacquinod, J. R. Johnson, D. Kouach, G. Lacombe, N. Lanza, L. Lapauw, J. Laserna, J. Lasue, L. le Deit, S. le Mouélic, E. le Comte, Q. M. Lee, C. Legett IV, R. Leveille, E. Lewin, C. Leyrat, G. Lopez-Reyes, R. Lorenz, B. Lucero, J. M. Madariaga, S. Madsen, M. Madsen, N. Mangold, F. Manni, J. F. Mariscal, J. Martinez-Frias, K. Mathieu, R. Mathon, K. P. McCabe, T. McConnochie, S. M. McLennan, J. Mekki, N. Melikechi, P. Y. Meslin, Y. Micheau, Y. Michel, J. M. Michel, D. Mimoun, A. Misra, G. Montagnac, C. Montaron, F. Montmessin, J. Moros, V. Mousset, Y. Morizet, N. Murdoch, R. T. Newell, H. Newsom, N. Nguyen Tuong, A. M. Ollila, G. Orttner, L. Oudda, L. Pares, J. Parisot, Y. Parot, R. Pérez, D. Pheav, L. Picot, P. Pilleri, C. Pilorget, P. Pinet, G. Pont, F. Poulet, C. Quantin-Nataf, B. Quertier, D. Rambaud, W. Rapin, P. Romano, L. Roucayrol, C. Royer, M. Ruellan, B. F. Sandoval, V. Sautter, M. J. Schoppers, S. Schröder, H. C. Seran, S. K. Sharma, P. Sobron, M. Sodki, A. Sournac, V. Sridhar, D. Standarovsky, S. Storms, N. Striebig, M. Tatat, M. Toplis, I. Torre-Fdez, N. Toulemont, C. Velasco, M. Veneranda, D. Venhaus, C. Virmontois, M. Viso, P. Willis, K. W. Wong The SuperCam instrument suite on the Mars 2020 rover: Science objectives and mast-unit description. *Space Sci. Rev.* **217**, 47 (2021).

52. R. C. Wiens, S. Maurice, S. H. Robinson, A. E. Nelson, P. Cais, P. Bernardi, R. T. Newell, S. Clegg, S. K. Sharma, S. Storms, J. Deming, D. Beckman, A. M. Ollila, O. Gasnault, R. B. Anderson, Y. André, S. Michael Angel, G. Arana, E. Auden, P. Beck, J. Becker, K. Benzerara, S. Bernard, O. Beyssac, L. Borges, B. Bousquet, K. Boyd, M. Caffrey, J. Carlson, K. Castro, J. Celis, B. Chide, K. Clark, E. Cloutis, E. C. Cordoba, A. Cousin, M. Dale, L. Deflores, D. Delapp, M. Deleuze, M. Dirmyer, C. Donny, G. Dromart, M. George Duran, M. Egan, J. Ervin, C. Fabre, A. Fau, W. Fischer, O. Forni, T.

Fouchet, R. Fresquez, J. Frydenvang, D. Gasway, I. Gontijo, J. Grotzinger, X. Jacob, S. Jacquinod, J. R. Johnson, R. A. Klisiewicz, J. Lake, N. Lanza, J. Laserna, J. Lasue, S. le Mouélic, C. Legett IV, R. Leveille, E. Lewin, G. Lopez-Reyes, R. Lorenz, E. Lorigny, S. P. Love, B. Lucero, J. M. Madariaga, M. Madsen, S. Madsen, N. Mangold, J. A. Manrique, J. P. Martinez, J. Martinez-Frias, K. P. McCabe, T. H. McConnochie, J. M. McGlown, S. M. McLennan, N. Melikechi, P. Y. Meslin, J. M. Michel, D. Mimoun, A. Misra, G. Montagnac, F. Montmessin, V. Mousset, N. Murdoch, H. Newsom, L. A. Ott, Z. R. Ousnamer, L. Pares, Y. Parot, R. Pawluczyk, C. Glen Peterson, P. Pilleri, P. Pinet, G. Pont, F. Poulet, C. Provost, B. Quertier, H. Quinn, W. Rapin, J. M. Reess, A. H. Regan, A. L. Reyes-Newell, P. J. Romano, C. Royer, F. Rull, B. Sandoval, J. H. Sarrao, V. Sautter, M. J. Schoppers, S. Schröder, D. Seitz, T. Shepherd, P. Sobron, B. Dubois, V. Sridhar, M. J. Toplis, I. Torre-Fdez, I. A. Trettel, M. Underwood, A. Valdez, J. Valdez, D. Venhaus, P. Willis The SuperCam instrument suite on the NASA Mars 2020 rover: Body unit and combined system tests. *Space Sci. Rev.* **217**, 4 (2021).

53. J. F. Bell, J. N. Maki, G. L. Mehall, M. A. Ravine, M. A. Caplinger, Z. J. Bailey, S. Brylow, J A Schaffner, K. M. Kinch, M. B. Madsen, A. Winhold, A. G. Hayes, P. Corlies, C. Tate, M. Barrington, E. Cisneros, E. Jensen, K. Paris, K. Crawford, C. Rojas, L. Mehall, J. Joseph, J. B. Proton, N. Cluff, R. G. Deen, B. Betts, E. Cloutis, A. J. Coates, A. Colaprete, K. S. Edgett, B. L. Ehlmann, S. Fagents, J. P. Grotzinger, C. Hardgrove, K. E. Herkenhoff, B. Horgan, R. Jaumann, J. R. Johnson, M. Lemmon, G. Paar, M. Caballo-Perucha, S. Gupta, C. Traxler, F. Preusker, M. S. Rice, M. S. Robinson, N. Schmitz, R. Sullivan, M. J. Wolff, The Mars 2020 Perseverance rover mast camera zoom (Mastcam-Z) multispectral, stereoscopic imaging investigation. *Space Sci. Rev.* **217**, 24 (2021).

54. A. G. Hayes, P. Corlies, C. Tate, M. Barrington, J. F. Bell, J. N. Maki, M. Caplinger, M. Ravine, K. M. Kinch, K. Herkenhoff, B. Horgan, J. Johnson, M. Lemmon, G. Paar, M. S. Rice, E. Jensen, T. M. Kubacki, E. Cloutis, R. Deen, B. L. Ehlmann, E. Lakdawalla, R. Sullivan, A. Winhold, A. Parkinson, Z. Bailey, J. van Beek, P. Caballo-Perucha, E. Cisneros, D. Dixon, C. Donaldson, O. B. Jensen, J. Kuik, K. Lapo, A. Magee, M. Merusi, J. Mollerup, N. Scudder, C. Seeger, E. Stanish, M. Starr, M. Thompson, N. Turenne, K. Winchell Pre-flight calibration of the Mars 2020 rover Mastcam zoom (Mastcam-Z) multispectral, stereoscopic imager. *Space Sci. Rev.* **217**, 29 (2021).

55. P. P. Shirvani, N. R. Saxena, E. J. McCluskey Software-implemented EDAC protection against SEUs. *IEEE Trans. Reliab.* **49**, 273–284 (2000).

56. E. W. Knutsen, O. Witasse, B. Sanchez-Cano, M. Lester, R. F. Wimmer-Schweingruber, M. Denis, J. Godfrey, A. Johnstone, Galactic cosmic ray modulation at Mars and beyond measured with EDACs on Mars Express and Rosetta. *Astron. Astrophys.* **650**, A165 (2021).

57. B. Sánchez-Cano, O. Witasse, E. W. Knutsen, D. Meggi, S. Viet, M. Lester, R. F. Wimmer-Schweingruber, M. Pinto, R. Moissl, J. Benkhoff, H. Opgenoorth, U. Auster, J. de Brujine, P. Collins, G. de Marchi, D. Fischer, Y. Futaana, J. Godfrey, D. Heyner, M. Holmstrom, A. Johnstone, S. Joyce, D. Lakey, S. Martinez, D. Milligan, E. Montagnon, D. Müller, S. A. Livi, T. Prusti, J. Raines, I. Richter, D. Schmid, P. Schmitz, H. Svedhem, M. G. G. T. Taylor, E. Tremolizzo, D. Titov, C. Wilson, S. Wood, J. Zender Solar energetic particle events detected in the housekeeping data of the European Space Agency's Spacecraft Flotilla in the Solar System. *Space Weather* **21**, e2023SW003540 (2023).

58. S. Viet, E. W. Knutsen, F. Montmessin, O. Witasse, B. S. Cano, M. Lester, R. F. Wimmer-Schweingruber, "Detecting and investigating space weather events around Mars with EDAC counters" in *73rd International Astronautical Congress Proceedings* (International Astronautical Federation, 2022).

59. B. Zhang, S. Zhang, G. Shen, C. Tuo, X. Zhang, H. Zhang, L. Quan, C. Tian, D. Hou, P. Zhou, W. Ji Monitor of the single event upsets and linear energy transfer of space radiation on the Beidou navigation satellites. *Open Astron.* **32**, 20220206 (2023).

60. D. E. Larson, R. J. Lillis, C. O. Lee, P. A. Dunn, K. Hatch, M. Robinson, D. Glaser, J. Chen, D. Curtis, C. Tiu, R. P. Lin, J. G. Luhmann, B. M. Jakosky The MAVEN solar energetic particle investigation. *Space Sci. Rev.* **195**, 153–172 (2015).

61. D. Zhao, J. Guo, H. Huang, H. Lin, Y. Hong, X. Feng, J. Cui, Y. Wei, Y. Wang, Y. Feng, L. Li, L. Liu Interplanetary coronal mass ejections from MAVEN orbital observations at Mars. *Astrophys. J.*,**923**, 4 (2021).

62. J. F. Bell 3rd, J. N. Maki, S. Alwmark, B. L. Ehlmann, S. A. Fagents, J. P. Grotzinger, S. Gupta, A. Hayes, K. E. Herkenhoff, B. H. N. Horgan, J. R. Johnson, K. B. Kinch, M. T. Lemmon, M. B. Madsen, J. I. Núñez, G. Paar, M. Rice, J. W. Rice Jr, N. Schmitz, R. Sullivan, A. Vaughan, M. J. Wolff, A. Bechtold, T. Bosak, L. E. Duflot, A. G. Fairén, B. Garczynski, R. Jaumann, M. Merusi, C. Million, E.

Ravanis, D. L. Shuster, J. Simon, M. S. Clair, C. Tate, S. Walter, B. Weiss, A. M. Bailey, T. Bertrand, O. Beyssac, A. J. Brown, P. Caballo-Perucha, M. A. Caplinger, C. M. Caudill, F. Cary, E. Cisneros, E. A. Cloutis, N. Cluff, P. Corlies, K. Crawford, S. Curtis, R. Deen, D. Dixon, C. Donaldson, M. Barrington, M. Ficht, S. Fleron, M. Hansen, D. Harker, R. Howson, J. Huggett, S. Jacob, E. Jensen, O. B. Jensen, M. Jodhpurkar, J. Joseph, C. Juarez, L. C. Kah, O. Kanine, J. Kristensen, T. Kubacki, K. Lapo, A. Magee, M. Maimone, G. L. Mehall, L. Mehall, J. Mollerup, D. Viúdez-Moreiras, K. Paris, K. E. Powell, F. Preusker, J. Proton, C. Rojas, D. Sallurday, K. Saxton, E. Scheller, C. H. Seeger, M. Starr, N. Stein, N. Turenne, J. Van Beek, A. G. Winhold, R. Yingling, Geological, multispectral, and meteorological imaging results from the Mars 2020 Perseverance rover in Jezero crater. *Sci. Adv.* **8**, eabo4856 (2022).

63. C. Legett, R. T. Newell, A. L. Reyes-Newell, A. E. Nelson, P. Bernardi, S. C. Bender, O. Forni, D. M. Venhaus, S. M. Clegg, A. M. Ollila, P. Pilleri, V. Sridhar, S. Maurice, R. C. Wiens Optical calibration of the SuperCam instrument body unit spectrometers. *Appl. Opt.* **61**, 2967–2974 (2022).

64. A. Kramida, Y. Ralchenko, NIST Atomic Spectra Database, NIST Standard Reference Database 78, 1999.

65. I. Laszlo, K. Stammes, W. J. Wiscombe, S.-C. Tsay, "The discrete ordinate algorithm, DISORT for radiative transfer" in *Light Scattering Reviews, Volume 11*, A. Kokhanovsky, Ed. (Springer, 2016), pp. 3–65.

66. D. P. Simonelli, M. Wisz, A. Switala, D. Adinolfi, J. Veverka, P. C. Thomas, P. Helfenstein Photometric properties of phobos surface materials from viking images. *Icarus* **131**, 52–77 (1998).

67. C. M. Ernst, R. T. Daly, R. W. Gaskell, O. S. Barnouin, H. Nair, B. A. Hyatt, M. M. al Asad, K. K. W. Hoch High-resolution shape models of phobos and deimos from stereophotoclinometry. *Earth Planets Space* **75**, 103 (2023).

68. R. T. Clancy, M. D. Smith, F. Lefèvre, T. H. Mc Connochie, B. J. Sandor, M. J. Wolff, S. W. Lee, S. L. Murchie, A. D. Toigo, H. Nair, T. Navarro, Vertical profiles of Mars 1.27 μm O_2 dayglow from MRO CRISM limb spectra: Seasonal/global behaviors, comparisons to LMDGCM simulations, and a global definition for Mars water vapor profiles. *Icarus* **293**, 132–156 (2017).

69. M. D. Smith, M. J. Wolff, R. T. Clancy, A. Kleinböhl, S. L. Murchie Vertical distribution of dust and water ice aerosols from CRISM Limb-geometry observations. *J. Geophys. Res. Planets* **118**, 321–334 (2013).

70. L. Soret, F. González-Galindo, J. C. Gérard, I. R. Thomas, B. Ristic, Y. Willame, A. C. Vandaele, B. Hubert, F. Lefèvre, F. Daerden, M. R. Patel Ultraviolet NO and visible O_2 nightglow in the Mars southern winter polar region: Statistical study and model comparison. *J. Geophys. Res. Planets*, **129**, e2024JE008620 (2024).

71. A. Migliorini, G. Piccioni, J. C. Gérard, L. Soret, T. G. Slanger, R. Politi, M. Snels, P. Drossart, F. Nuccilli The characteristics of the O_2 Herzberg II and chamberlain bands observed with VIRTIS/Venus Express. *Icarus*, **223**, 609–614 (2013).

72. M. Dierckxsens, K. Tziotziou, S. Dalla, I. Patsou, M. S. Marsh, N. B. Crosby, O. Malandraki, G. Tsiropoula, Relationship between solar energetic particles and properties of flares and CMEs: Statistical analysis of solar cycle 23 events. *Sol. Phys.* **290**, 841–874 (2015).

73. S. A. Haider, K. K. Mahajan, S. W. Bougher, N. M. Schneider, J. Deighan, S. K. Jain, J. C. Gérard Observations and modeling of martian auroras. *Space Sci. Rev.* **218**, 32 (2022).

74. B. Sanchez-Cano, the M-MATISSE team, *The M-MATISSE mission: Mars Magnetosphere ATmosphere Ionosphere and Space weather SciencEAn ESA Medium class (M7) candidate*.

75. M. T. Lemmon, S. D. Guzewich, T. McConnochie, A. de Vicente-Retortillo, G. Martínez, M. D. Smith, J. F. Bell III, D. Wellington, S. Jacob Large dust aerosol sizes seen during the 2018 martian global dust event by the Curiosity rover. *Geophys. Res. Lett.* **46**, 9448–9456 (2019).

76. M. J. Wolff, M. D. Smith, R. T. Clancy, R. Arvidson, M. Kahre, F. Seelos IV, S. Murchie, H. Savijärvi, Wavelength dependence of dust aerosol single scattering albedo as observed by the compact reconnaissance imaging spectrometer. *J. Geophys. Res.* **114**, E00D04 (2009).
